# Supplementary material for: Mapping the Scientific Landscape of Bacterial Influence on Oral Cancer: A Bibliometric Analysis of the Last Decade’s Medical Progress
Source: Curr Oncol. 2023 Oct 5;30(10):9004–18. doi: 10.3390/curroncol30100650 (PMC10604929; doi:10.3390/curroncol30100650)
Supplement: Supplementary file 1 [file curroncol-30-00650-s001.zip › curroncol-2627833-supplementary.pdf]

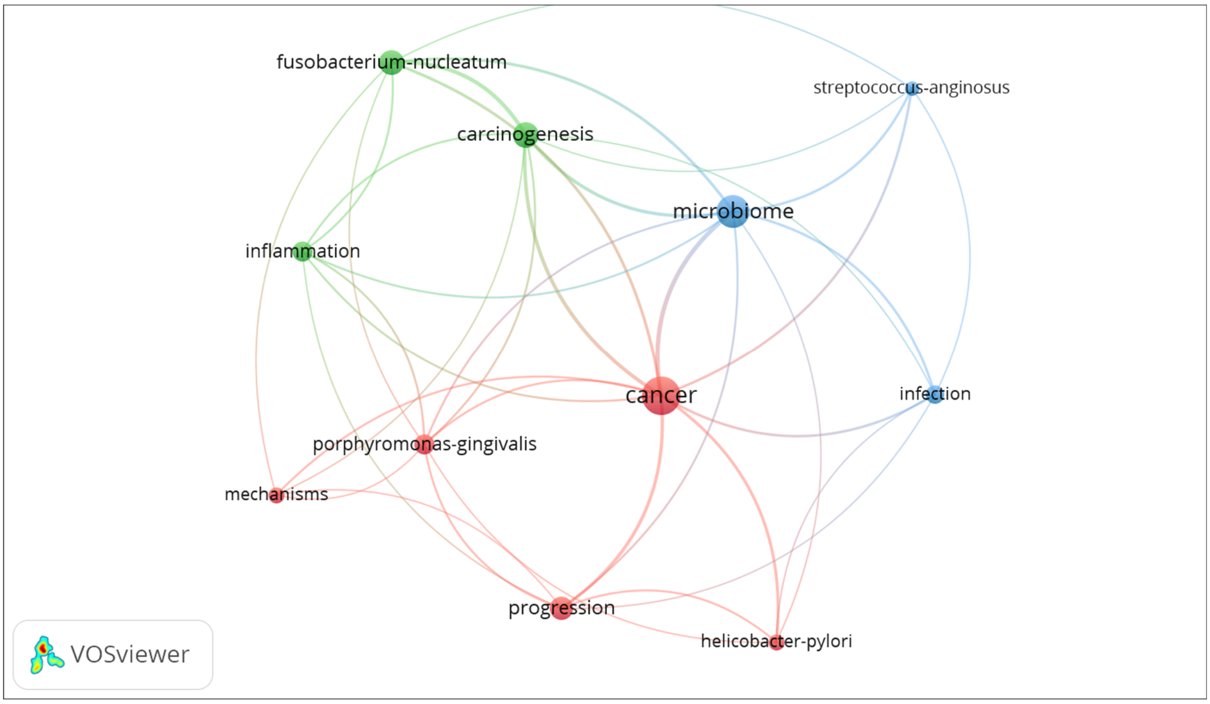

Figure S1. Co-occurrence network of bacterium-associated keywords of relevant publications.

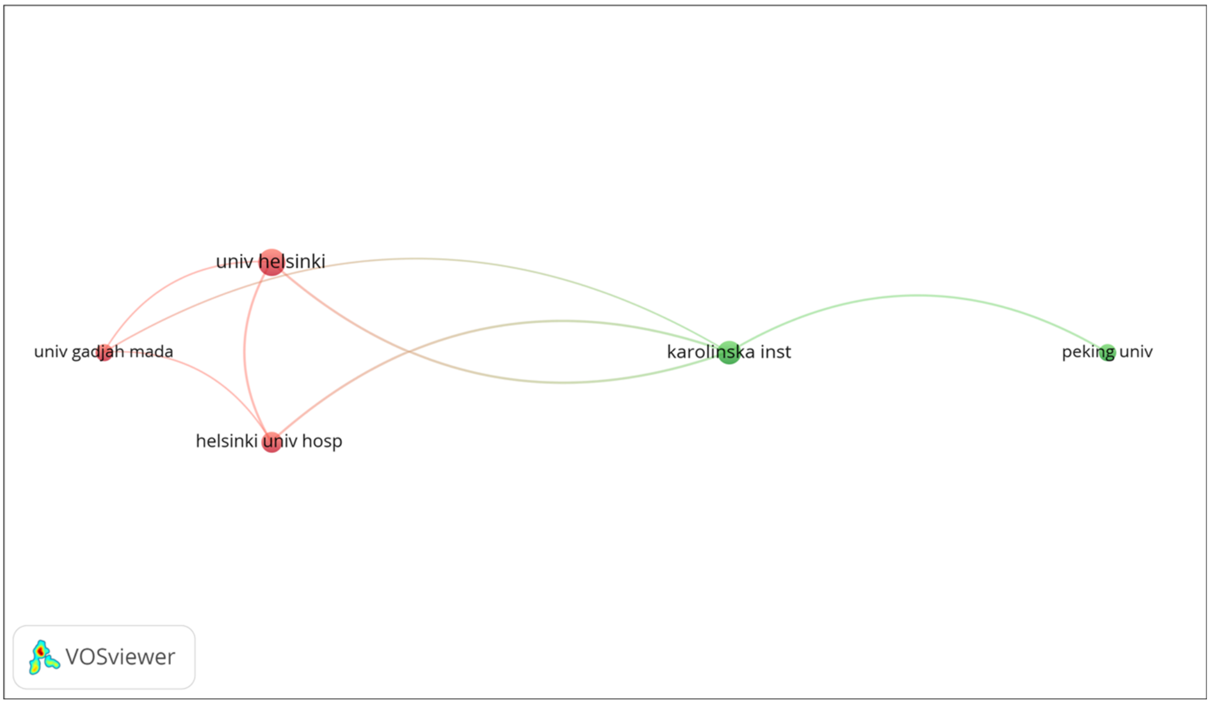

Figure S2. Co-authorship network of different affiliations.
